# Expression profiles of carnosine synthesis–related genes in mice after ingestion of carnosine or ß-alanine

**DOI:** 10.1186/1550-2783-9-15

**Published:** 2012-04-17

**Authors:** Takayuki Miyaji, Mikako Sato, Hirohiko Maemura, Yoshihisa Takahata, Fumiki Morimatsu

**Affiliations:** 1 Research and Development Center, Nippon Meat Packers, Inc., 3-3 Midorigahara, Tsukuba, Ibaraki, 300-2646, Japan; 2 Department of Physical Education, International Pacific University, 721 Kannonji seto-cho, Higashi-ku, Okayama, 709-0863, Japan

**Keywords:** Carnosine, ß-alanine, Carnosine synthase, Carnosinase

## Abstract

**Background:**

Carnosine is a dipeptide that improves exercise performance. The carnosine synthesis mechanism through carnosine and ß-alanine ingestion remains unclear. Therefore, we investigated the tissue distribution of carnosine synthase, ATP-grasp domain-containing protein-1 (ATPGD1) mRNA, and ATPGD1 and carnosine specific dipeptidase (CN1) gene expression profiles in mice that were given carnosine or ß-alanine orally.

**Methods:**

ddY mice (7-week-old) were randomly divided into three groups (n = 6 to 8 animals per group) and were orally given 2 g/kg body weight of carnosine, ß-alanine, or water. After 15, 30, 60, 120, 180, or 360 min of treatment, the tissues (brain, blood, liver, kidneys, olfactory bulbs, hindleg muscles) were collected. The obtained tissues measured the expression of ATPGD1 and CN1 genes using quantitative PCR methods.

**Results:**

The ATPGD1 gene was expressed in muscle and to a lesser extent in brain. The expression of ATPGD1 in the vastus lateralis muscle increased significantly at 180 min (*P* = 0.023) after carnosine ingestion and 60 (*P* = 0.023) and 180 min (*P* = 0.025) after ß-alanine ingestion. Moreover, the carnosine group showed a significantly increased renal expression of the CN1 gene 60 min after ingestion (*P* = 0.0015).

**Conclusions:**

The ATPGD1 gene showed high expression levels in brain and muscle. The ß-alanine or carnosine administration significantly increased ATPGD1 and CN1 expression in mice.

## Background

Carnosine (ß-alanyl-L-histidine) is a dipeptide abundant in mammalian skeletal muscles [1,2]. Various physiological actions have been ascribed to carnosine in muscle, including acting as an antioxidant [3], regulating Ca^2+^ sensitivity [4], protecting proteins against glycation by acting as a sacrificial peptide [5], and preventing the formation of protein–protein cross links by reacting with protein-carbonyl groups [6]. Primarily, carnosine with pH buffering capacity is widely used in the field of sports nutrition [7]. Because the dissociation exponent (pKa) of carnosine is 6.83 [8,9], it is suggested that carnosine attenuates the reduction in blood pH by a large amount of H^+^ originating from the dissociation of lactic acid during strenuous exercise, and suppresses a loss of force [10]. At the same time, muscle carnosine contents are positively correlated with high-intensity exercise performance [11] and fast-twitch muscle fibers [12]. Increase of muscle carnosine predominantly was due to the ingestion of histidine-containing dipeptide (HCD) such as carnosine, anserine (ß-alanyl-1-methylhistidine) and balenine (ß-alanyl-3-methylhistidine) or ß-alanine. Although ß-alanine could also be synthesized from the degradation of uracil, there are no reports on the relation between carnosine synthesis and pyrimidine catabolism. So, the majority of the previous research relating to the ergogenic effects of elevated muscle carnosine content via chicken breast extract, high in HCD content or ß-alanine supplementation was performed using mice, horses and humans [13-17].

However, ingested carnosine is rapidly degraded by two forms of carnosinase (CN1, EC 3.4.13.20; and CN2, EC 3.4.13.18) [18]. In humans, the CN1 gene is expressed in liver and brain tissue, and the protein is found in serum and brain tissue. Since the human CN1 specifically degrades both carnosine and homocarnosine, carnosine is absent in human blood. Whereas, CN1 in other mammals such as rodents is localized in the kidney, and a considerable amount of carnosine is contained in the blood [19]. CN2, which is also a cytosolic non-specific dipeptidase, does not degrade homocarnosine, and exhibits a rather broad specificity towards various dipeptides. That is, ingestion of ß-alanine or carnosine that was degraded by these carnosinases, was increased muscle carnosine and the increase of muscle carnosine may be involved in carnosine synthase. However, the details were not revealed.

Recently, carnosine synthase was purified from chicken pectoral muscle and identified as an ATP-grasp domain-containing protein 1 (ATPGD1) [20]. It has been reported that ATPGD1 synthesizes carnosine using ATP, and the substrate specificity toward ß-alanine (carnosine) in the presence of ATP and L-histidine is 14-fold higher than that of γ-aminobutyrate (homocarnosine). To verify that ATPGD1 acts as a carnosine synthase *in vivo*, we investigated the tissue distribution of ATPGD1 mRNA, and ATPGD1 and CN1 expression profiles in response to carnosine or ß-alanine administration using quantitative PCR analysis.

## Methods

### Oral administration study in mice

Animal experiments were performed in accordance with the guidelines for Animal Experiments at Nippon Meat Packers Inc. and using minimum number of mice that dictated by an ethics committee ( n = 6 or 8). Male SPF-bred ddY (6-week-old) mice were purchased from Japan SLC, Inc. (Shizuoka, Japan). The mice were maintained under specifically controlled environmental conditions, namely, a 12-h light–dark cycle, a temperature of 23°C, and a relative humidity of 50%. At 7 weeks of age, the mice were randomly assigned by body weight into three groups (pre-administration group, n = 8, body weight of 32.5 g; water administration group, n = 6, body weight of 33.4 g; carnosine administration group, n = 6 or 8, body weight of 33.2 g; ß-alanine administration group, n = 6, body weight of 34.0 g) and were orally given 2 g/kg body weight of carnosine (Hamari Chemicals Ltd., Osaka, Japan), ß-alanine (Wako Pure Chemical Industries, Ltd., Osaka, Japan), or water (control). After 15, 30, 60, 120, 180, or 360 min of treatment, the mice were anesthetized with Forane (Abbott Japan Co. Ltd., Japan) and then the brain, blood, liver, kidneys, olfactory bulbs, soleus muscles and vastus lateralis muscles were collected. The collected tissues were weighed, rapidly frozen with liquid nitrogen, and stored at −80°C until analysis.

### Extraction of total RNA

The frozen tissue samples were homogenized in 0.75 ml of Isogen (Nippon Gene Co. Ltd., Tokyo, Japan) and then mixed thoroughly with 0.15 ml of chloroform. The mixture was centrifuged (20,000 × *g* for 5 min), and then the aqueous phases were collected, and 0.4 ml of isopropanol was added. The precipitated total RNA was recovered and washed with 70% (v/v) ethanol. The purity and concentration of the total RNA thus obtained were confirmed using an Experion electrophoresis system (Bio-Rad Laboratories, Inc., California, USA) and a NanoDrop 1000 spectrophotometer (Thermo Fisher Scientific K. K., Massachusetts, USA).

### Construction of gene specific primers

Gene specific primers were designed by using Primer-BLAST (http://www.ncbi.nlm.nih.gov/tools/primer-blast/). The primers used were as follows: for ATPGD1 (NM_134148), forward primer, 5′-CCCTGGCCTTCGACCTCTCTCCAT-3′ and reverse primer, 5′-CGGCACTGGGGCCCATCCTTC-3′ to yield a 164-bp product; for CN1 (NM_177450), forward primer, 5′-TGGTGGCATCCTCAACGAACCA-3′ and reverse primer, 5′-TCCAGGAATTAGGATGTGGCCTGA-3′ to yield an 88-bp product; for ß-actin (NM_007393), forward primer, 5′-ATGAGCTGCCTGACGGCCAGGTCATC-3′ and reverse primer, 5′-TGGTACCACCAGACAGCACTGTGTTG-3′ to yield a 192-bp product.

### Quantification of mRNA levels

cDNA was synthesized by using a PrimeScript RT reagent Kit with gDNA Eraser (Takara Bio, Inc., Shiga, Japan). The genomic DNA in the RNAs extracted from tissues was eliminated with gDNA Eraser, which were then reverse-transcribed by PrimeScript RT. Each 25 μl of the PCR reaction mix contained a 2 μl template, 0.2 μM of each primer, and 1× ROX Reference Dye II in 1× SYBR Premix Ex Taq II (Takara Bio, Inc.). The reaction was performed at 95°C for 30 s; this was followed by 40 cycles at 95°C for 5 s and at 60°C for 20 s. The fluorescence was measured at the end of the extension step in each cycle. Following cycling, a melt curve analysis was performed after each quantitative PCR to ensure that a single product had been amplified per primer set. The fold-change of the gene expression was calculated using the 2^-∆∆Ct^ method with ß-actin as an internal control. Student’s *t*-test was used (*P* < 0.05 or *P* < 0.01) to test statistical significance.

### Detection of carnosine in muscle and blood

Vastus lateralis muscle samples were deproteinized with 1 ml of 5% (w/v) sulfosalicylic acid. The samples were centrifuged at 20,000 × *g* for 5 min, and then the supernatants were filtered with a 0.45-μm filter. Blood samples were dissolved in 1 M perchloric acid (final concentration, 0.3 M) and centrifuged at 20,000 × *g* for 5 min. KOH (3 M) was added to the supernatants to realize a final concentration of 4.25% v/v. After centrifugation (20,000 × *g* for 5 min), the obtained supernatants were filtered and applied to a TSKgel ODS-80Ts column (Tosoh Co., Tokyo, Japan) equilibrated with 4% (v/v) acetonitrile, 100 mM sodium 1-pentanesulfonate, and 200 mM ammonium dihydrogen phosphate (pH 2.0). The carnosine was eluted with the same buffer, and absorbance was detected at a wavelength of 214 nm. Statistical analysis was performed with Tukey-Kramer test (*P* < 0.05 or *P* < 0.01).

## Results

### Tissue distribution of ATPGD1 mRNA

**Figure 1:**
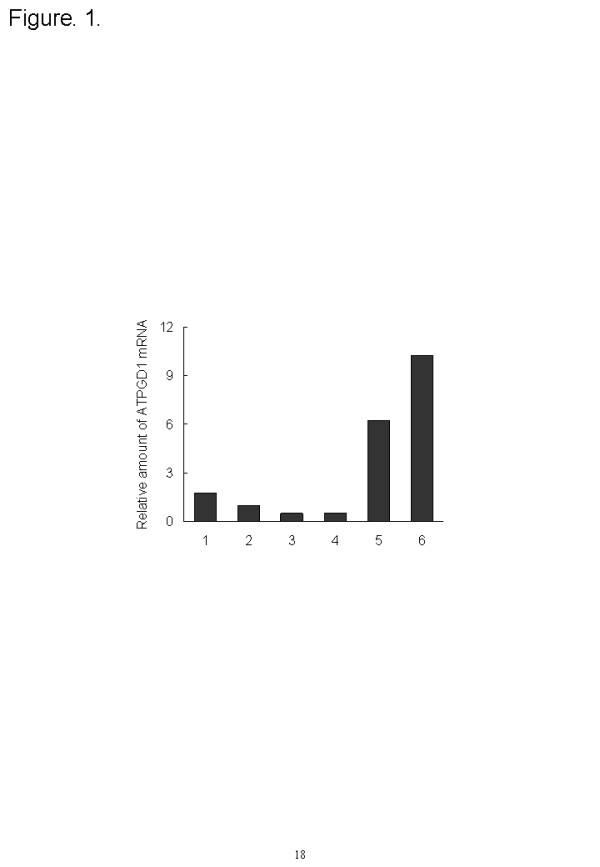
**Tissue distribution of ATPGD1 mRNA in mice.** 1; brain, 2; olfactory bulbs, 3; kidneys, 4; liver, 5; soleus muscles, and 6; vastus lateralis muscles. ß-actin gene (Actb) was used as an endogenous control gene.

The localization of ATPGD1 mRNA from various tissue samples was investigated by quantitative PCR methods. ATPGD1 genes were detected in muscle, a few in brain, and hardly in liver and kidney. The expression of ATPGD1 was 10.2-fold higher in the vastus lateralis muscle, 6.3-fold higher in the soleus muscle and 1.8-fold higher in the brain than in the olfactory bulbs. In contrast, the expression of ATPGD1 in the liver and kidney was only 50% of that in the olfactory bulbs (Figure 1).

### Carnosine content of blood and muscle

**Figure 2:**
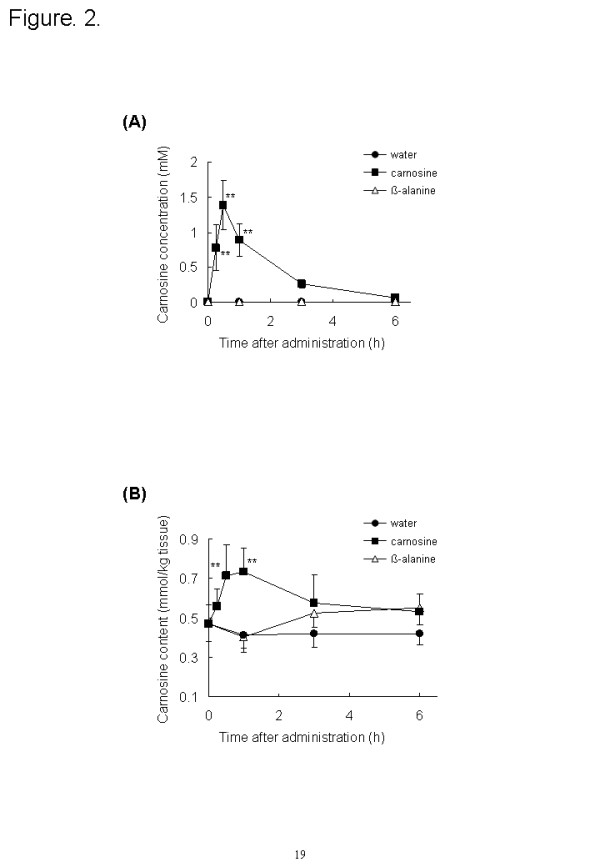
**Time course of carnosine concentration in blood (A), vastus lateralis muscles (B) and following ingestion of carnosine, ß-alanine, or water; 2 g/kg body weight carnosine (closed squares), ß-alanine (open triangles), or water (closed circles) was orally administered to mice (n = 6–8).** Values are means ± SD. Significant differences after administration were analyzed by using Tukey-Kramer test (**P < 0.01).

In mice that had ingested carnosine or ß-alanine, we measured the carnosine content of the blood and vastus lateralis muscle by using an ODS-80Ts column. The carnosine content of the blood had significantly increased by 15 min after carnosine administration (*P* < 0.01); it peaked at 30 min (1.4 ± 0.3 mM, *P* < 0.01) and had nearly disappeared by 6 h (Figure 2A). No carnosine was detected in the blood of the groups that ingested ß-alanine or water. As shown Figure 2B, the carnosine content of the vastus lateralis muscle was 0.47 ± 0.09 mmol/kg tissue before administration. The carnosine level had increased significantly 30 to 60 min after it was administered (0.71 ± 0.15 mmol/kg tissue at 30 min, *P* < 0.01 and 0.74 ± 0.12 mmol/kg tissue at 60 min, *P* < 0.01) and then gradually decreased. The carnosine content of muscle in the group that ingested ß-alanine did not increase significantly compared with that before administration (*P* > 0.05).

### Gene expression of ATPGD1 and CN1

**Figure 3:**
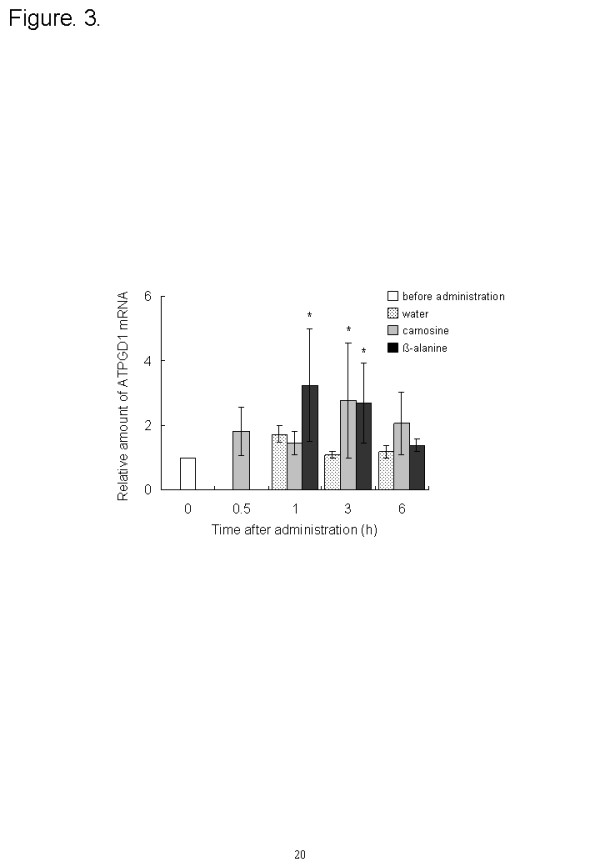
**Effect of dietary carnosine and ß-alanine on ATPGD1 mRNA expression in the vastus lateralis muscle of male mice; 2 g/kg body weight of carnosine, ß-alanine, or water was orally administered to mice (n = 6–8). Values are means ± SD.** Significant differences after administration were analyzed by using Student’s *t*-test (* *P* < 0.05).

**Figure 4:**
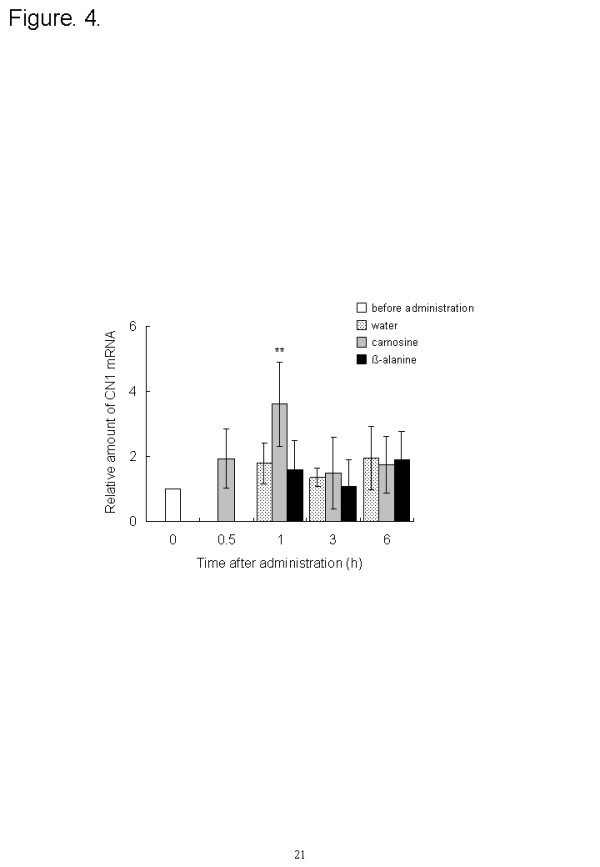
**Effect of dietary carnosine and ß-alanine on the CN1 mRNA expression in the kidneys of male mice; 2 g/kg body weight of carnosine, ß-alanine, or water was orally administered to mice (n = 6–8). Values are means ± SD.** Significant differences after administration were analyzed by using Student’s t-test (**P < 0.01).

The expression profiles of carnosine synthesis-related genes were measured by using quantitative PCR. The ATPGD1 mRNA level in the vastus lateralis muscle was significantly elevated 3 h after carnosine administration (*P* = 0.023) and at 1 (*P* = 0.023) and 3 h (*P* = 0.025) after ß-alanine administration, compared with the level before administration. Expression increased from 2.7 to 3.2 times that before ingestion (Figure 3). After carnosine ingestion, the CN1 expression in the kidney peaked at 1 h and was significantly greater (3.6 times, *P* = 0.0015) than before ingestion (Figure 4).

## Discussion

Carnosine synthase have been tried to purify from various sources [21-24] and Drozak et al. purified carnosine synthase from chicken pectoral muscle and the enzyme identified as ATPGD1, which is a member of the ATP-grasp family [20]. This paper was investigated about whether ATPGD1 involved in carnosine synthesis in mice.

Firstly, the tissue distribution of the ATPGD1 gene was investigated. The ATPGD1 gene was expressed more in brain and muscle than in olfactory bulbs, liver and kidney and particularly in the vastus lateralis muscle. The expression of the ATPGD1 gene was 1.6-fold higher than that in the soleus muscle. The carnosine content in the vastus lateralis muscle (0.47 mmol/kg tissue) was higher than in the soleus muscle (0.35 mmol/kg tissue, *P* = 0.007, data not shown), indicating that the ATPGD1 mRNA level depends on the carnosine content.

Secondly, we investigated the carnosine content and the expression of carnosine synthesis-related genes after the ingestion of carnosine or ß-alanine. The carnosine supplementation group increased the carnosine content in blood and muscle and the expression of CN1 in the kidneys. Carnosine was injected into the tail vein of proton-coupled oligopeptide transporter PEPT2 knockout mice and the kidney/plasma concentration ratio of carnosine in the PEPT2 null mice was one-sixth that in wild-type [25]. Thus, it was considered that the ingested carnosine was eliminated from the serum by filtration into the urine and reabsorption into the kidney, and the reabsorbed carnosine increased the expression of CN1 in the kidney and would be hydrolyzed to ß-alanine. Carnosine and ß-alanine administration increased the ATPGD1 gene levels in the vastus lateralis muscles. This suggests that the hydrolyzed ß-alanine in kidney increased ATPGD1 gene expression. Recently, Baguet et al. investigated the expression of ATPGD1 mRNA in human skeletal muscle. Twenty omnivorous subjects were randomly divided into a vegetarian and a mixed diet group, and took part in a five-week sprint training intervention (2–3 times per week). The ATPGD1 mRNA expression in the vegetarian diet group was decreased to 60 % (*P* = 0.023) by five weeks of sprint training [26]. This is consistent with our result showing that ß-alanine is an important factor in ATPGD1 expression.

Chronic chicken breast extract or ß-alanine supplementation leads to improved performance in high-intensity exercise [27,28]. However, the loading of carnosine takes at least several weeks [29], in contrast to the initial loading phase of one week for creatine [30]. This paper suggests that ATPGD1 acts as a carnosine synthase in mice, and provides new insights to determine efficient muscle carnosine loading.

## Conclusions

The present study shows that the ATPGD1 mRNA in mice was expressed highly in brain and muscle, moderately in olfactory bulbs, scarcely in liver and kidneys, and approximately 67 mg of ß-alanine or carnosine administration in mice significantly increased ATPGD1 and CN1 expression.

## Competing interests

The authors declare that they have no competing interests.

## Authors' contributions

TM is the principal investigator of the project. MS, HM, YT and FM designed the study; MS and HM collected the data; YT and FM conducted data analysis; TM, MS and HM wrote the manuscript. All authors have read and approved the final manuscript.
